# Exposure to second-hand smoke is an independent risk factor of small airway dysfunction in non-smokers with chronic cough: A retrospective case-control study

**DOI:** 10.3389/fpubh.2022.912100

**Published:** 2022-07-20

**Authors:** Bingrong Zhao, Lu Bai, Rongjun Wan, Yanan Wang, Ling Qin, Qiming Xiao, Pinhua Pan, Chengping Hu, Juan Jiang

**Affiliations:** ^1^Department of Respiratory Medicine, National Key Clinical Specialty, Branch of National Clinical Research Center for Respiratory Disease, Xiangya Hospital, Central South University, Changsha, China; ^2^Center of Respiratory Medicine, Xiangya Hospital, Central South University, Changsha, China; ^3^Clinical Research Center for Respiratory Diseases in Hunan Province, Changsha, China; ^4^Hunan Engineering Research Center for Intelligent Diagnosis and Treatment of Respiratory Disease, Changsha, China; ^5^National Clinical Research Center for Geriatric Disorders, Xiangya Hospital, Changsha, China

**Keywords:** small airway dysfunction, second-hand smoke, chronic cough, non-smoker, gender, environmental tobacco smoke

## Abstract

**Objectives:**

This study aimed to identify the potential risk factors for small airway dysfunction (SAD) in non-smokers with chronic cough.

**Methods:**

Non-smokers with chronic cough who underwent lung function tests at Xiangya Hospital from May 2019 to May 2020 were enrolled, and divided into the derivation and validation cohorts based on their hospital admission time. SAD was determined based on the presence of at least two of the following three indicators of lung function being less than 65% of predicted: maximal mid-expiratory flow, forced expiratory flow at 50% of forced vital capacity (FVC), and forced expiratory flow at 75% of FVC. Clinical data of these patients were collected. Risk factors for SAD were identified by logistic regression analysis in the derivation cohort and further confirmed in the validation cohort.

**Results:**

In total, 316 patients (152 in the non-SAD group and 164 in the SAD group) were included in the derivation cohort. Compared with the non-SAD group, the SAD group had a higher proportion of female patients (82.3 vs. 59.2%, *P* < 0.001), was more commonly exposed to second-hand smoke (SHS) (61.6 vs. 27.6%, *P* < 0.001), and tended to be older (median age, 45.5 vs. 40.0 years old, *P* = 0.004). The median FVC, forced expiratory volume in one second (FEV_1_) % pred, FEV_1_/FVC ratio, and peak expiratory flow (PEF) % pred were slightly lower in the SAD group. Multivariable logistic analysis showed that exposure to SHS was an independent risk factor (OR 4.166 [95% CI 2.090–8.302], *P* < 0.001) for SAD in non-smokers with chronic cough after adjusting for related variables. In the validation cohort (*n* = 146), patients with SHS exposure had a relative risk of 1.976 (95% CI 1.246–3.135, *P* = 0.004) for SAD compared to those without SHS exposure. Multivariable logistic analysis consistently confirmed that exposure to SHS was an independent risk factor (OR 3.041 [95% CI 1.458–6.344], *P* = 0.003) for SAD in non-smokers.

**Conclusions:**

Exposure to SHS is independently associated with a higher risk of SAD in non-smokers with chronic cough. Reduction in SHS exposure may ameliorate lung function, thus lowering the risk of irreversible airway obstruction.

## Introduction

Chronic cough is the most common symptom in outpatients with respiratory disorders. In China, patients with chronic cough account for more than one-third of respiratory outpatients, most of whom are never-smokers. Persistent cough symptoms seriously impair the quality of life of these patients and pose a huge burden on public health resources (1–3). Approximately 30–50% of patients with chronic cough exhibit eosinophil infiltration and aggregation, which further leads to the development of airway inflammation (4). A considerable proportion of patients with chronic cough do not reach the stage of asthma or chronic obstructive pulmonary disease (COPD), but lung function tests suggest the presence of small airway dysfunction (SAD) (5).

Small airways are defined as those with a lumen diameter <2 mm (6). According to a recent national cross-sectional study, more than 40% of adults have SAD in China (5). An increasing number of studies have confirmed that SAD is closely related to a multitude of respiratory diseases and can be a precursor sign of asthma and COPD that appears before pulmonary radiological changes (7, 8). Moreover, SAD is associated with asthma severity and progression. Individuals presenting with obvious symptoms usually reach the stage of irreversible lung function impairment (9). Therefore, early identification and effective intervention for SAD in patients with chronic cough are of clinical significance to avoid further damage to lung function.

Previous studies have identified multiple risk factors for SAD, including smoking status, age, gender, urbanization, and education level (5, 10). However, risk factors for SAD in the non-smoking population with chronic cough as the chief complaint remain unclear. Therefore, a retrospective case-control study was conducted to summarize the clinical features of non-smokers with chronic cough and to explore the potential risk factors for SAD.

## Methods

### Study design and subjects

**Figure 1:**
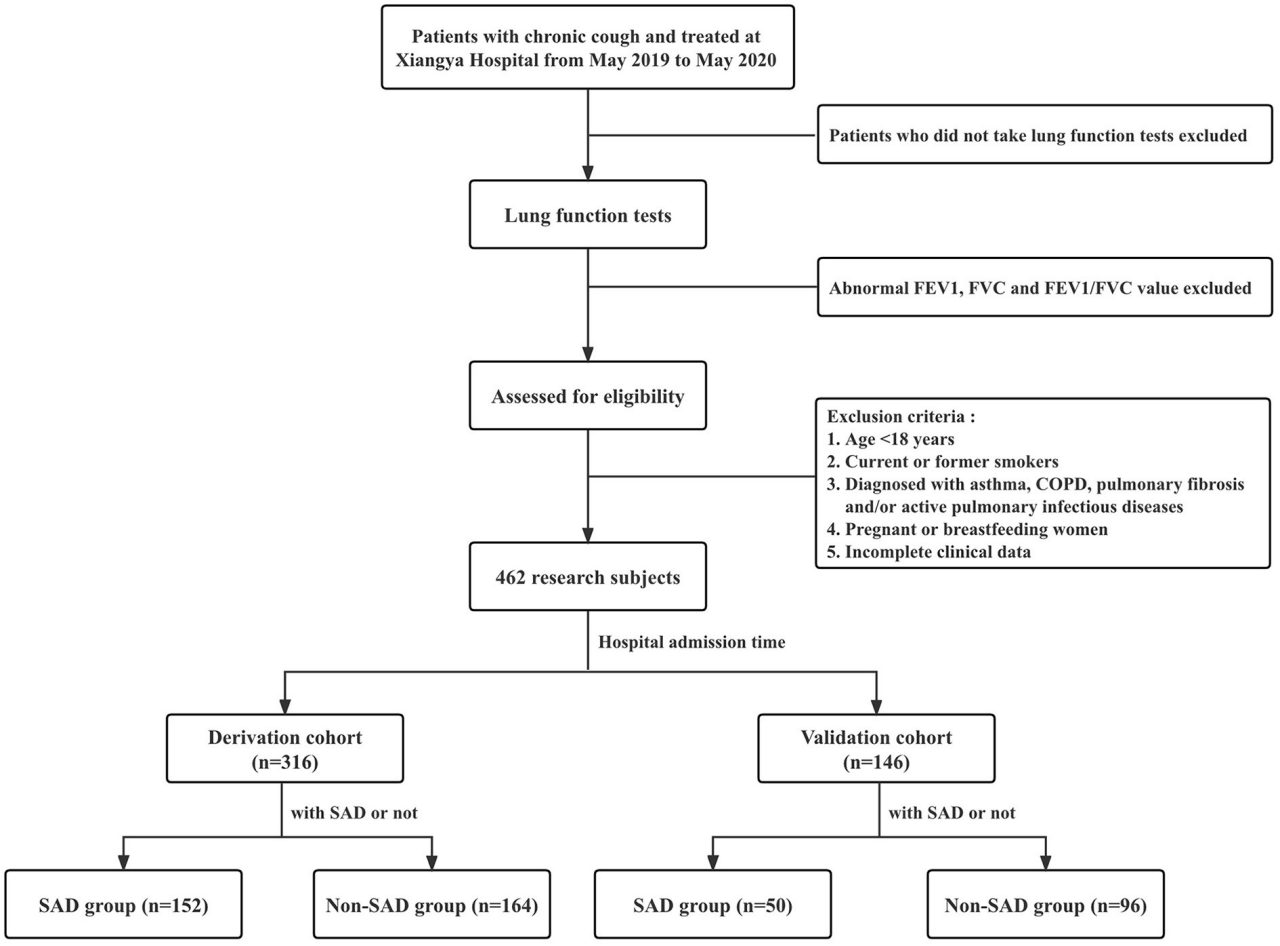
Flowchart showing patient enrollment in this study. FEV_1_, forced expiratory volume in one second; FVC, forced vital capacity; FEV_1_/FVC, the ratio of forced expiratory volume in one second to forced vital capacity; COPD, chronic obstructive pulmonary disease; SAD, small airway dysfunction; bpm, beats per minute.

A flowchart of patient enrollment is shown in Figure 1. In this retrospective case-control study, patients with chronic cough who attended the Department of Respiratory Medicine, Xiangya Hospital, Central South University from May 1 2019 to May 31 2020 were consecutively included. Patients who met all of the following criteria were eligible for enrollment: (1) patients with cough as the only or main symptom and a disease duration > 8 weeks (11); (2) patients who underwent lung function tests; (3) patients with normal FVC, FEV_1_ and FEV_1_/FVC ratio; and (4) age ≥ 18 years old. Patients who met any of the following criteria were excluded: (1) age <18 years; (2) current or former smokers; (3) patients who were diagnosed with asthma, COPD, pulmonary fibrosis and/or active pulmonary infectious diseases; (4) patients who were pregnant or breastfeeding; and (5) incomplete clinical data. Enrolled patients were divided into the non-SAD and SAD groups according to the presence or absence of SAD. Eligible patients admitted to our hospital because of chronic cough between May 2019 and December 2019 were included in the derivation cohort to explore the potential risk factors for SAD. Eligible patients admitted to our hospital between January 2020 and May 2020 were included in the validation cohort to further validate the risk factors found in the derivation cohort.

### Lung function tests

All participants underwent lung function tests performed by trained technicians using a Jaeger MasterScreen pneumotachograph spirometer (CareFusion, Yorba Linda, CA, USA). Each participant was required to perform up to eight forced expiratory maneuvers until the FVC and FEV_1_ were reproducible within 150 mL. All spirometry data were reviewed by an expert panel based on the criteria of the American Thoracic Society and European Respiratory Society (12). A normal FVC or FEV_1_ was defined as no less than 80% of the predicted value, and a normal FEV_1_/FVC ratio was defined as no less than 92% of the predicted value based on reference values in the Chinese population (13).

### Data collection

Demographics, history of respiratory diseases, exposure to second-hand smoke (SHS), laboratory findings, and lung function parameters of each patient were obtained from medical records. Demographics included age, gender, and body mass index (BMI) of patients. Exposure to SHS was defined as exposure to tobacco smoke as a result of having one or more active smokers who regularly smoked cigarettes at home and in the workplace and/or entertainment venues (14). Information on exposure to SHS in different environments was obtained through a direct query. Laboratory indicators included peripheral blood cell count, liver function, renal function, serum immunoglobulin E (IgE) level, and allergen skin tests. Lung function parameters included FVC, maximal mid-expiratory flow (MMEF) % pred, forced expiratory flow (FEF) 50% pred, FEF75% pred, FEV_1_/FVC ratio, FEV_1_, peak expiratory flow (PEF), fractional exhaled nitric oxide (FeNO).

### Statistical analysis

R software (version 3.6.3, www.r-project.org) was used for data analysis. Continuous variables with normal distribution are presented as mean ± standard deviation and compared using Student's *t*-test, while continuous variables with non-normal distribution are expressed as median (interquartile ranges) and compared using the Mann–Whitney *U* test. Categorical variables are presented as counts and percentages and compared using the chi-square test or Fisher's exact test. Wilcoxon rank-sum and signed-rank tests were used to compare lung function in the non-SAD and SAD groups. Univariable logistic regression analysis was used to identify potential risk factors for SAD in non-smokers with chronic cough, and variables that showed statistical significance were selected for inclusion in a multivariable logistic regression analysis to further define independent risk factors after adjusting for potential confounders. The interaction effect between two different variables was assessed using the lrm function in the R package rms to fit the logistic regression model with maximum likelihood estimation. The Wald test was used to test the interaction effect. Relative risk was calculated by dividing the probability of SAD in group with exposure to SHS by the probability of SAD in group without exposure to SHS. *P*-values <0.05 were considered statistically significant.

## Results

### Demographics and baseline clinical features in the derivation cohort

In total, 316 patients were enrolled in the derivation cohort. Of these patients, 152 and 164 were divided into the non-SAD and SAD groups based on the absence or presence of SAD, respectively. Demographic characteristics, history of respiratory diseases, and laboratory findings of all patients are shown in Table 1. Overall, the median age was 44.0 years, and the mean BMI was 23.06 kg/m^2^. Among them, 225 (71.2%) were female, and 91 (28.8%) were male. History of respiratory diseases included chronic bronchitis (8.5%), tuberculosis (17.7%), bronchiectasis (3.5%), and interstitial lung disease (1.9%). Compared with the non-SAD group, the proportion of females was higher (82.3% vs. 59.2%, *P* < 0.001) and patients tended to be older (median age, 45.5 vs. 40.0 years, *P* = 0.004) in the SAD group. No significant differences were found in laboratory findings between the two groups, including peripheral white blood cell counts, hemoglobin, liver function, renal function, serum IgE, and allergen skin tests.

**Table 1:** Demographics, pre-existing respiratory disease and laboratory indicators of the derivation cohort.

**Variables** **[*****n*** **(%), median** **(IQR) or mean** ±**SD]**	**Total** **(*****n** =* **316)**	**Non-SAD group** **(*****n** =* **152)**	**SAD group** **(*****n** =* **164)**	***P*** **value**
**Age, years**	**44.0 (31.0, 52.0)**	**40.0 (27.7, 52.0)**	**45.5 (36.0, 52.0)**	**0.004**
**Gender**			
Male Female	91 (28.8) 225 (71.2)	62 (40.8) 90 (59.2)	29 (17.7) 135 (82.3)	<0.001
BMI (kg/m^2^)	23.06 ± 3.36	22.82 ± 3.46	23.28 ± 3.25	0.217
Exposure to SHS	143 (45.3)	42 (27.6)	101 (61.6)	<0.001
Respiratory disease history Chronic bronchitis Pulmonary tuberculosis Bronchiectasis ILD	100 (31.6) 27 (8.5) 56 (17.7) 11 (3.5) 6 (1.9)	45 (29.6) 12 (7.9) 24 (15.8) 7 (4.6) 2 (1.3)	55 (33.5) 15 (9.1) 32 (19.5) 4 (2.4) 4 (2.4)	0.190 0.824 0.442 0.458 0.499
WBC (× 10^9^/L)	6.31 ± 1.61	6.31 ± 1.40	6.46 ± 1.79	0.393
Neutrophil (× 10^9^/L)	3.50 (2.80, 4.20)	3.50 (3.00, 3.98)	3.50 (2.70, 4.65)	0.825
Lymphocyte (× 10^9^/L)	1.97 ± 0.59	1.92 ± 0.67	2.02 ± 0.52	0.498
Eosinophil (× 10^9^/L)	0.10 (0.10, 0.20)	0.10 (0.02, 0.20)	0.10 (0.10, 0.20)	0.209
Hemoglobin (g/L)	134.0 (127.0, 151.0)	136.0 (126.2, 156.7)	133.0 (127.5, 143.5)	0.306
Serum albumin (g/L)	45.60 ± 4.33	44.98 ± 2.40	46.08 ± 5.41	0.578
TBil (μmol/L)	12.60 (9.00, 14.90)	11.50 (7.20, 13.70)	13.30 (10.15, 15.23)	0.355
SCr (μmol/L)	74.63 ± 11.25	76.01 ± 12.23	73.78 ± 11.03	0.671
BUN (mmol/L)	4.09 ± 0.92	4.08 ± 0.92	4.10 ± 0.95	0.952
Serum IgE (IU/mL)	148.20 (19.25, 360.25)	172.30 (35.65, 510.00)	82.85 (16.12, 229.20)	0.345
Positive allergen skin test	9 (2.8)	3 (2.0)	6 (3.7)	0.504

*IQR, interquartile ranges; SD, standard deviation; SAD, small airway dysfunction; BMI, body mass index; ILD, interstitial lung disease; WBC, white blood cell; TBil, total bilirubin; SCr, serum creatine; BUN, blood urea nitrogen; IgE, immunoglobulin E*.

### Lung function parameters in the derivation cohort

**Figure 2:**
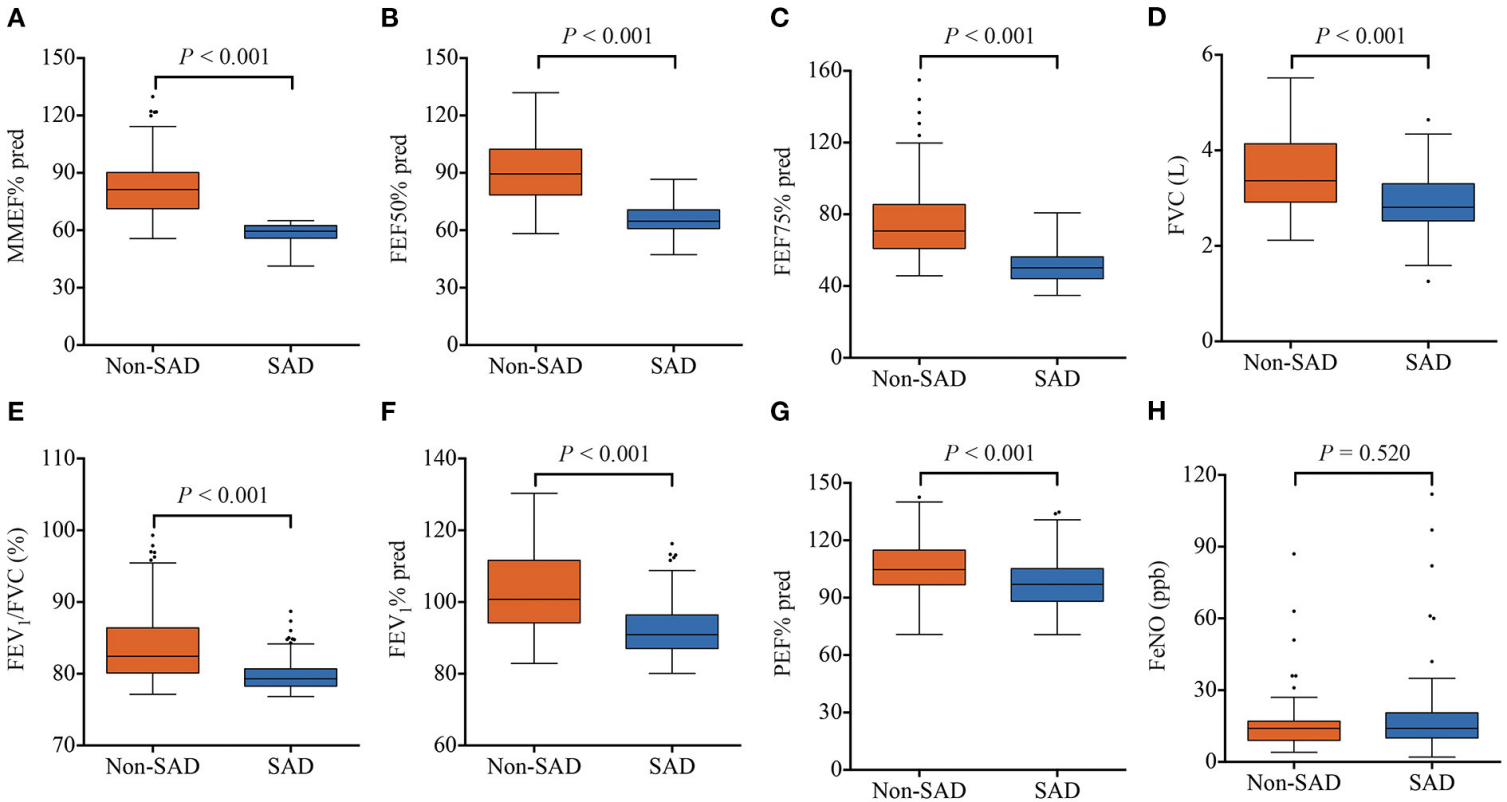
Lung function parameters of the SAD and non-SAD groups in the derivation cohort. **(A)** MMEF% pred; **(B)** FEF50% pred; **(C)** FEF75% pred; **(D)** FVC; **(E)** FEV_1_/FVC; **(F)** FEV_1_% pred; **(G)** PEF% pred; **(H)** FeNO. Orange squares represent the non-SAD group and blue squares represent the SAD group. SAD, small airway dysfunction; MMEF, maximum mid-respiratory flow; FEF 50%, forced expiratory flow at 50% of forced vital capacity; FEF 75%, forced expiratory flow at 75% of forced vital capacity; FEV_1_/FVC, the ratio of forced expiratory volume in one second to forced vital capacity; FEV_1_, forced expiratory volume in one second; PEF, peak expiratory flow rate; FeNO, fractional exhaled nitric oxide.

Lung function parameters of all patients were analyzed. As expected, patients in the SAD group showed impaired small airway function parameters, including lower MMEF% pred (59.55 vs. 81.30%, *P* < 0.001), FEF50% pred (64.75 vs. 89.50%, *P* < 0.001), and FEF75% pred (50.20 vs. 70.75%, *P* < 0.001), compared with those in the non-SAD group (Figures 2A–C). Interestingly, median FVC (2.81 vs. 3.37 L, *P* < 0.001), FEV_1_% pred (90.95 vs. 100.75%, *P* < 0.001), FEV_1_/FVC ratio (79.31 vs. 82.44%, *P* < 0.001), and PEF% pred (97.51 vs. 106.16%, *P* < 0.001) were mildly lower in the SAD group (Figures 2D–G). There were 208 patients who underwent FeNO detection. As shown in Figure 2H, FeNO values were similar between the two groups (*P* = 0.520).

### Exposure to SHS in the derivation cohort

**Figure 3:**
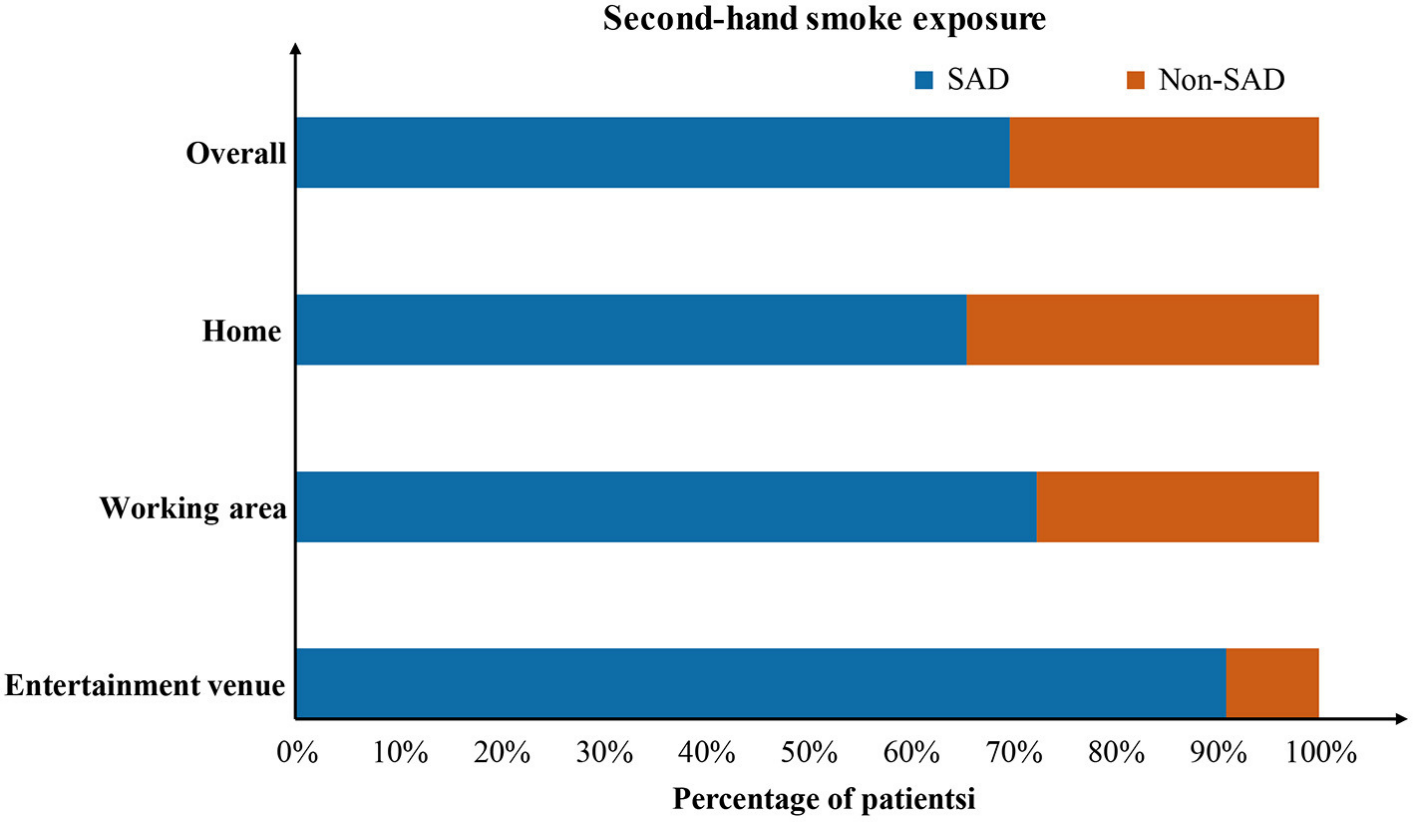
Exposure to second-hand smoke of the SAD and non-SAD groups in the derivation cohort. The orange squares represent the non-SAD group, and the blue squares represent the SAD group.

The proportion of patients who were exposed to SHS was significantly higher (61.6 vs. 27.6%, *P* < 0.001) in the SAD group than in the non-SAD group, as shown in Table 1. Further tracing the environments of SHS exposure (Figure 3), the results showed that more patients in the SAD group had SHS exposure in different environments, including home, workplace, and entertainment venues. Next, SHS exposure was compared between male and female patients. As shown in Supplementary Figure 1, there was a higher proportion of female patients who were exposed to SHS than male patients (50.8 vs. 23.8%, *P* = 0.002).

### Risk factors of SAD in non-smokers with chronic cough

To identify potential risk factors for SAD in non-smokers with chronic cough, a univariable logistic regression analysis was conducted. As shown in Table 2, SHS exposure, female gender, and age were potentially associated with SAD in non-smokers. In the multivariable logistic regression analysis (Table 3), our data showed that SHS exposure was an independent risk factor for SAD in non-smokers with chronic cough after adjusting for age and female gender (OR 4.174 [95% CI 2.127–8.193], *P* < 0.001) and after adjusting for age, female gender, BMI, and history of respiratory diseases (OR 4.166 [95% CI 2.090–8.302], *P* < 0.001). Female gender was independently associated with a higher risk of SAD after adjusting for age and exposure to SHS (OR 2.348 [95% CI 1.067–5.167], *P* = 0.034), which was not observed after additionally adjusting for BMI and history of respiratory diseases (OR 2.164 [95% CI 0.939–4.987], *P* = 0.070). No significant interaction effect was found between SHS exposure and female gender (*P* = 0.766) in logistic regression.

**Table 2:** Univariable logistic regression analysis of risk factors associated with SAD in the derivation cohort.

**Variables**	**OR**	**95% CI**	***P*** **value**
Age	1.027	1.010–1.044	0.002
Gender (= female)	3.172	1.895–5.308	<0.001
BMI	1.044	0.977–1.115	0.207
Exposure to SHS	4.776	2.483–9.185	<0.001
History of chronic bronchitis	1.199	0.525–2.739	0.666
History of pulmonary tuberculosis	1.250	0.275–5.678	0.773
History of bronchiectasis	0.521	0.150–1.818	0.307
History of ILD	0.932	0.130–6.700	0.944
White blood cell	1.159	0.859–1.563	0.335
Neutrophil	1.129	0.773–1.649	0.530
Lymphocyte	1.374	0.619–3.050	0.435
Eosinophil	7.988	0.386–16.16	0.179
Hemoglobin	0.989	0.971–1.008	0.262
Serum albumin	1.066	0.856–1.327	0.569
Total bilirubin	1.056	0.947–1.178	0.330
Serum creatine	0.984	0.909–1.066	0.698
Serum IgE	0.998	0.994–1.003	0.435
Positive allergen skin test	1.899	0.466–7.729	0.371

*OR, odds ratio; CI, confidence interval; SAD, small airway dysfunction; BMI, body mass index; SHS, second-hand smoke; ILD, interstitial lung disease; IgE, immunoglobulin E*.

**Table 3:** Multivariable logistic regression analysis of risk factors associated with SAD in the derivation cohort.

	**Model 1**		**Model 2**	
**Variables**	**OR (95% CI)**	***P*** **value**	**OR (95% CI)**	***P*** **value**
Exposure to SHS	4.174 (2.127–8.193)^a^	<0.001	4.166 (2.090–8.302)^c^	<0.001
Gender (= female)	2.348 (1.067–5.167)^b^	0.034	2.164 (0.939–4.987)^d^	0.070

*^a^Adjusted for age and gender*;

*^b^adjusted for age and exposure to SHS*;

*^c^adjusted for age, gender, BMI, and history of respiratory diseases*.

*^d^adjusted for age, exposure to SHS, BMI, and history of respiratory diseases. OR, odds ratio; CI, confidence interval; SAD, small airway dysfunction*.

### Validation of the association between SHS exposure and SAD

The association between SHS exposure and SAD was further confirmed in patients of the validation cohort, whose demographic and clinical characteristics were summarized in Supplementary Table 1. As shown in Table 4, patients with SHS exposure had a relative risk of 1.976 (95% CI 1.246–3.135, *P* = 0.004) for the outcome of SAD, which supports and validates the results obtained in the derivation cohort. In the multivariable logistic regression analysis (Table 5), our data consistently demonstrated that SHS exposure was an independent risk factor of SAD in non-smokers with chronic cough after adjusting for age and female gender (OR 3.160 [95% CI 1.525–6.550], *P* = 0.002), or after additionally adjusting for age, female gender, BMI and history of respiratory diseases (OR 3.041 [95% CI 1.458–6.344], *P* = 0.003).

**Table 4:** Relative risk of SHS exposure for SAD in the validation cohort.

	**Outcomes**		
**Risk factor**	**Non-SAD**	**SAD**	**Relative risk (95% CI)**	***P*** **value**
Exposure to SHS				
No	63	20	1	
Yes	33	30	1.976 (1.246–3.135)	0.004

*SHS, second-hand smoke; CI, confidence interval; SAD, small airway dysfunction*.

**Table 5:** Odds ratios of SHS exposure on multivariable logistic regression analysis for SAD in the validation cohort.

	**Model 1**		**Model 2**	
**Variables**	**OR (95% CI)**	***P*** **value**	**OR (95% CI)**	***P*** **value**
Exposure to	3.160 (1.525–6.550)^a^	0.002	3.041 (1.458–6.344)^b^	0.003
SHS				

*^a^Adjusted for age and gender*;

*^b^adjusted for age, gender, BMI, and history of respiratory diseases. SHS, second-hand smoke; OR, odds ratio; CI, confidence interval; SAD, small airway dysfunction*.

## Discussion

Herein, we conducted a retrospective study to analyze the clinical characteristics of non-smoking patients presenting with chronic cough, and explore the risk factors for SAD in this subpopulation. This study is the first to demonstrate that SHS exposure is an independent risk factor for SAD in non-smokers with chronic cough.

Small airways, known as the “silent zone” of the lung disease, have a large surface area and strong reserve capacity. SAD is recognized as a precursor to chronic airway diseases such as asthma and COPD (8, 15). From a structural point of view, the total volume and collective surface area of distal airways greatly exceed those of central airways (16). Small airways contain little or no cartilage and are easily collapsible, making them the predominant site of airflow resistance (17). From a functional point of view, SAD leads to premature airway closure, air trapping and peripheral heterogeneity of ventilation, which significantly contributes to increased airflow resistance in patients with chronic airway diseases (18). From a cellular perspective, cells of the small airway epithelium, ciliated cells and goblet cells undergo various morphological and/or functional alterations, eventually resulting in airway remodeling. Moreover, increased immune cell infiltration in the small airways, including macrophages, neutrophils and T lymphocytes, correlates with the degree of airflow obstruction and contributes to lung damage in chronic airway diseases (19–21). More importantly, SAD occurs earlier than tissue destruction, as well as clinically detectable airflow limitation. It does not usually cause clinical symptoms and is often overlooked (22, 23). Proper small airway diagnostic assessment in routine clinical practice with early recognition of SAD is essential for lowering the risk of developing chronic airway diseases. While many studies consider cigarette smoking as a risk factor for SAD and enroll smokers as participants, it is worth noting that the majority of patients presenting with chronic cough are lifelong non-smokers (24, 25). Therefore, the present study focused on non-smokers with chronic cough as the main symptom, with the purpose of analyzing the potential risk factors for SAD in this subpopulation.

One of the most interesting findings of the present study is that SHS exposure emerges as an risk factor for SAD in non-smokers with chronic cough. SHS is defined as tobacco smoke produced by an active smoker, both from the exhalation of smoked tobacco and the burning end of the cigarette, that is inhaled by nonsmokers (26, 27). Previous studies have reported that smoking is associated with an increased risk of SAD (28). However, most studies have focused on the effects of active smoking on small airway function (29), ignoring the dangers of exposure to SHS. As research has evolved, the damage caused by SHS exposure on lung function has been confirmed and may even play a role in the progression to chronic airway diseases such as asthma (30, 31). In this study, multivariable logistic regression analysis confirmed that SHS exposure was independently associated with a higher risk of SAD in non-smokers with chronic cough after adjusting for potentially related variables, indicating that more attention should be paid to patients with SAD who are exposed to SHS, especially in non-smokers presenting with chronic cough. As early as 1980, a study suggested that passive smoking may reduce small airway function, and that the effects vary with the degree of smoke exposure (32). Exposure to SHS leads to an increase in the number of circulating neutrophils and increased chemotaxis (33), which also activates the release of inflammatory factors, such as interleukin-17A (34), thus promoting an inflammatory response. Moreover, continuous exposure to SHS disrupts the balance between Th1 and Th2 cells in the lungs (35), promoting the aggregation of Th2 cells and production of inflammatory factors, which in turn promote inflammatory responses. In addition, previous studies have confirmed that the small airways are the main site of inflammatory effects of cigarette smoke in humans (36). Therefore, we speculate that SHS exposure can activate the production of multiple inflammatory factors that promote the inflammatory response of the small airways and may promote the development of SAD. Currently, SHS exposure is a critical global public health issue. It is estimated that 600,000 individuals die each year owing to the effects of SHS (37). Our study showed links between SHS exposure and SAD, further emphasizing the importance of reducing SHS exposure to improve lung health. Since the World Health Organization Framework Convention on Tobacco Control was signed, exposure to SHS among non-smokers has gradually declined in China from 2010 to 2015; nevertheless, it remains a serious issue. Based on our data, SHS exists in various surrounding environments, including home, working areas and entertainment venues. A considerable proportion of patients in this study were exposed to SHS in public spaces, which highlights the necessity of banning active smoking in public spaces. Introducing a legislative smoking ban (especially in public spaces), spreading awareness about the hazards of SHS, and increasing tobacco prices and taxes can be effective ways to reduce SHS exposure (38, 39).

Existing evidence on the correlation between gender and SAD is lacking so far. Our data suggest that female gender might be associated with SAD in non-smokers with chronic cough. It has been reported that lung function is influenced by female sex hormones (40), and androgens affect lung function by inhibiting lung surfactant production in a variety of species through mechanisms that involve altering epidermal growth factor and transforming growth factor-β signaling (41). However, whether these factors ultimately affect small airway function remains unclear. In addition, long-term exposure to cooking oil fumes may be related to SAD in traditional Chinese women. In China, women play a leading role in domestic cooking. Therefore, in a typical domestic context, women have several periods per day of intense exposure to cooking oil fumes. It has been reported that 85.0% of women reported cooking at home (42); these women are at risk of exposure to toxic compounds from the burning of fuel and fumes when cooking. Different cooking methods, especially frying, emit large amounts of PM 2.5 (43), and exposure to PM 2.5 increases the risk of SAD (5), further highlighting the importance of reducing the burden of home cooking for women. Other effective interventions include the use of environmentally friendly fuels, improved ventilation systems and cooking technology (44).

In the present study, patients in the SAD group had slightly but statistically significantly lower FVC, FEV_1_% pred, FEV_1_/FVC ratio, and PEF% pred than those in the non-SAD group. This suggests that the non-smokers with chronic cough combined with SAD may present with a trend of airflow obstruction, which in turn supports that SAD is an early stage of chronic airway diseases. Previous studies have demonstrated that FeNO levels negatively correlate with small airway indexes in non-smokers, rather than smokers (45, 46). In our study, no significant association was observed between FeNO and SAD in non-smokers. This may be explained by the fact that almost half of the non-smokers in our study were exposed to SHS.

The present study have several limitations. First, this is a single-center retrospective study with a relatively small sample size, thus inherent bias and confounding effects of unrecognized factors are unavoidable. This could be an important reason why the association between female gender and SAD was not clearly determined in logistic regression. Second, quantification of exposure to SHS was not performed, which presents a line of inquiry worthy to pursue in future studies. Finally, long-term follow-up of these patients is lacking to observe the dynamic changes in lung function parameters, especially small airway function parameters. Large-scale prospective studies are required to determine how the frequency, duration and category of SHS exposure affect small airway function and whether small airway function parameters can be improved by avoiding exposure to SHS in non-smokers.

## Conclusions

Exposure to SHS is an independent risk factors for SAD in non-smokers with chronic cough. Therefore, it is important to conduct lung function tests in non-smokers with chronic cough. Reduction or avoidance of SHS exposure may be beneficial for improving small airway function, thus lowering the risk of chronic airway diseases.

## Data availability statement

The raw data supporting the conclusions of this article will be made available by the authors, without undue reservation.

## Ethics statement

The studies involving human participants were reviewed and approved by the Ethics Committee of Xiangya Hospital, Central South University (Changsha, Hunan, China). Written informed consent for participation was not required for this study in accordance with the national legislation and the institutional requirements.

## Author contributions

JJ and BZ: study concept and design and critical revision of the manuscript. BZ, LB, RW, YW, LQ, QX, PP, and CH: acquisition of data. RW and LB: statistical analysis. JJ, BZ, and LB: analysis and interpretation of data. JJ and LB: drafting of the manuscript. All authors contributed to the article and approved the submitted version.

## Funding

This work was supported by grants from the National Key Technology R&D Program of China (2015BAI12B10), National Natural Science Foundation of China (82100099 and 82170041), and the Innovative Research Platform of Hunan Development and Reform Commission (2021-212).

## Conflict of interest

The authors declare that the research was conducted in the absence of any commercial or financial relationships that could be construed as a potential conflict of interest.

## Publisher's note

All claims expressed in this article are solely those of the authors and do not necessarily represent those of their affiliated organizations, or those of the publisher, the editors and the reviewers. Any product that may be evaluated in this article, or claim that may be made by its manufacturer, is not guaranteed or endorsed by the publisher.
