# Supplementary material for: Exposure to second-hand smoke is an independent risk factor of small airway dysfunction in non-smokers with chronic cough: A retrospective case-control study
Source: Front Public Health. 2022 Jul 20;10:912100. doi: 10.3389/fpubh.2022.912100 (PMC9347364; doi:10.3389/fpubh.2022.912100)
Supplement: Supplementary file 1 [file Table_1.DOCX]

**Supplementary table 1** Demographics, pre-existing respiratory disease and laboratory indicators of validation cohort

| Variables [n (%), median  (IQR) or mean ± SD] | Total  (n=146) | Non-SAD group (n=96) | SAD group  (n=50) | *P* value |
| --- | --- | --- | --- | --- |
| Age, years | 42.5 (30.0, 52.0) | 40.0 (29.0, 50.0) | 47.5 (35.0, 54.0) | 0.054 |
| Gender  Male  Female | 32 (21.9)  114 (78.1) | 22 (22.9)  74 (77.1) | 10 (20.0)  40 (80.0) | 0.847 |
| BMI (kg/m^2^) | 23.38±3.33 | 23.11±3.42 | 23.92±3.12 | 0.165 |
| Exposure to SHS | 63 (43.2) | 33 (34.4) | 30 (60.0) | 0.005 |
| Respiratory disease history  Chronic bronchitis  Pulmonary tuberculosis | 17 (11.6)  3 (2.1) | 11 (11.5)  3 (3.1) | 6 (12.0)  0 | 1.000  0.551 |
| WBC (×10^9^/L) | 6.28±1.58 | 6.21±1.66 | 6.45±1.41 | 0.638 |
| Neutrophil (×10^9^/L) | 3.75±1.40 | 3.76±1.48 | 3.71±1.26 | 0.905 |
| Lymphocyte (×10^9^/L) | 1.90±0.57 | 1.82±0.55 | 2.13±0.58 | 0.094 |
| Eosinophil (×10^9^/L) | 0.10 (0.10, 0.20) | 0.10 (0.02, 0.20) | 0.10 (0.10, 0.20) | 0.939 |
| Hemoglobin (g/L) | 137.35±17.31 | 136.61±15.95 | 139.23±20.97 | 0.648 |
| Serum albumin (g/L) | 46.7 (43.9-48.1) | 46.2 (43.7-47.8) | 46.7 (45.0-48.9) | 0.851 |
| TBil (μmol/L) | 10.47±3.98 | 10.49±3.70 | 10.38±5.59 | 0.956 |
| SCr (μmol/L) | 71.0 (64.4-80.0) | 71.1 (63.7-80.7) | 69.0 (68.7-73.0) | 0.772 |
| BUN (mmol/L) | 4.20 (3.59-5.23) | 4.14 (3.52-5.47) | 4.55 (4.28-4.91) | 0.934 |
| Positive allergen skin test | 6 (4.1) | 5 (5.2) | 1 (2.0) | 0.664 |

IQR, interquartile ranges; SD, standard deviation; SAD, small airway dysfunction; BMI, body mass index; WBC, white blood cell; TBil, total bilirubin; SCr, serum creatine; BUN, blood urea nitrogen.
