# Supplementary figures and images for: Exposure to second-hand smoke is an independent risk factor of small airway dysfunction in non-smokers with chronic cough: A retrospective case-control study
Source: Front Public Health. 2022 Jul 20;10:912100. doi: 10.3389/fpubh.2022.912100 (PMC9347364; doi:10.3389/fpubh.2022.912100)

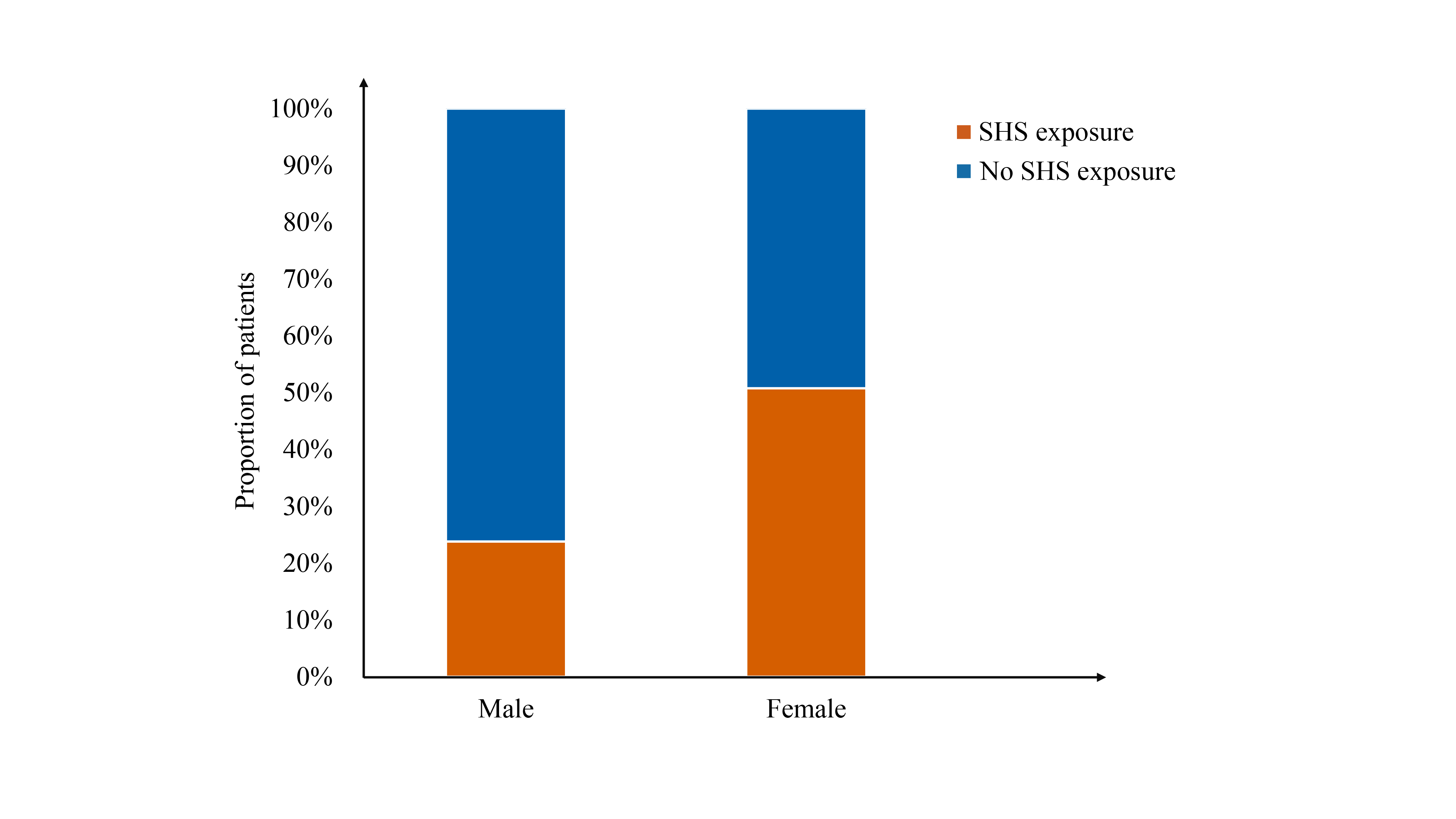

Supplement: Supplementary file 2 [file Image_1.jpeg]
